# Development of two antigen-binding fragments to a conserved linear epitope of human adenovirus and their application in immunofluorescence

**DOI:** 10.1371/journal.pone.0219091

**Published:** 2019-06-26

**Authors:** Zhenwei Liu, Xingui Tian, Wenkuan Liu, Yuting Xian, Weilue Chen, Huaying Chen, Rong Zhou

**Affiliations:** State Key Laboratory of Respiratory Disease, National Clinical Research Center for Respiratory Disease, Guangzhou Institute of Respiratory Health, The First Affiliated Hospital of Guangzhou Medical University, Guangzhou Medical University, Guangzhou, China; US Naval Research Laboratory, UNITED STATES

## Abstract

Detection of human adenoviruses (HAdVs) in nasopharyngeal swab samples by immunofluorescence assay (IFA) will be valuable for diagnosing HAdV infection, which is a leading cause of severe respiratory tract disease, and will help in curbing the spread of HAdV. Monoclonal antibodies employed in IFA for HAdV detection should ideally target highly conserved epitope types. Here, we describe the development of two antigen-binding fragments (Fabs) with specific reactivity to HAdV using phage antibody library technology. When tested with IFA, both Fabs recognized cells infected with several types of HAdV, some of which have been identified in epidemics globally, or associated with outbreaks of severe or fatal acute respiratory diseases. The specificity and cross-reactivity of both Fabs to HAdVs indicated that the generated Fabs could be applied in the development of IFAs to detect HAdVs. Both Fabs bound to the knob proteins, as shown by chemiluminescence enzyme immunoassay and western blot. In addition, epitope mapping showed that both Fabs recognized a conserved linear epitope among several types of HAdV. Two different Fabs recognized the same epitope, suggesting that the epitope triggered the production of at least two kinds of antibodies in the body. The generated Fabs exerted no neutralization against HAdVs. The results demonstrate that both Fabs bind to an epitope that plays no role in neutralization of HAdV.

## Introduction

Human adenovirus (HAdV) is a non-enveloped virus with an icosahedral shaped capsid that consists of three major proteins (fiber, hexon, and penton base proteins) and four minor proteins[1]. The fiber, hexon, and penton base proteins of HAdV have been shown to be the antigens that cause the body to produce antibodies[2]. The fiber protein is a trimeric complex composed of three domains: an N-terminus tail, a rod-like shaft, and a globular knob at the C-terminus[3]. The knob domain is responsible for specific high-affinity binding of HAdV to the cell receptor and determines HAdV infection and tissue tropism[4]. Since HAdV was first isolated by Rown *et al*. in 1953, 90 types have been identified so far (http://hadvwg.gmu.edu/), including candidates from HAdV type 53 (HAdV-53) to HAdV-90, and these are classified into seven species (from A to G) on the basis of serology, whole-genome sequencing, and phylogenomics[5, 6]. HAdVs are common pathogens that cause a variety of clinical diseases, such as acute respiratory disease (ARD), cystitis, gastroenteritis, and keratoconjunctivitis[6]. Among HAdVs associated with ARD, type 1 to type 7 HAdVs have been identified in epidemics globally and are recognized as the main pathogens involved[7–11]. Numerous outbreaks of severe or even fatal ARD caused by re-emergent HAdV-14 in species B (HAdV-B14) and HAdV-B55 have been reported in many countries over the last decade[12–14].

The diagnosis of HAdV infection has mainly been determind using culture-based techniques, antigen detection by immunofluorescence assay (IFA), and real-time PCR[15–17]. HAdV grows readily in cell culture, but culture-based techniques may require weeks to deliver definitive results[18]. IFA and real-time PCR offer advantages in terms of speed, and the diagnosis of HAdV infection can be completed within 2 h[16, 18]. Detection of HAdVs in nasopharyngeal swab samples by IFA is well-established and commonly used[19]. Monoclonal antibodies (mAbs) employed by IFA to detect HAdVs should ideally target highly conserved epitope types. A mAb found to be reactive against HAdVs in species C by binding with the hexon protein was reported for application in immunofluorescence, but the epitope was not further identified[16]. Here, we describe the development of two antigen-binding fragments (Fabs), named 2A6 and 4E2, with specific reactivity to HAdV using phage antibody library technology. We discuss the application of both Fabs in immunofluorescence techniques. In addition, epitope mapping showed that both Fabs recognized a conserved linear epitope among several types of HAdV.

## Materials and methods

### Ethics statement

The research was approved by the Ethics Committee at the First Affiliated Hospital of Guangzhou Medical University. Both informed and written consent was obtained from the participant who provided the sample.

### Viruses and cells

The viruses used in this study are listed in the Table 1. Different types of HAdV were separately purified using the ViraTrap Adenovirus Mini Purification Kit (Biomiga, San Diego, CA, USA), and the others viruses were separately purified using the ViraTrap Virus Mini Purification Kit (Biomiga, San Diego, CA, USA). A549 cells and AD293 cells were separately cultured in Dulbecco’s modified Eagle medium: nutrient mixture F-12 (DMEM/F-12) (GIBCO BRL, Grand Island, NY, USA) supplemented with 10% heat-inactivated (56 °C for 30 min) fetal bovine serum (GIBCO BRL, Grand Island, NY, USA) and antibiotics (100 U/mL penicillin, 100 μg/mL streptomycin) (GIBCO BRL, Grand Island, NY, USA) at 37 °C in 5% CO_2_.

**Table 1:** The viruses used in this study.

Virus	Strain	Culture cell	Reference
HAdV-B3	GZ01	A549	[20]
HAdV-B7	GZ08	A549	[21]
HAdV-B11	Slobitski	A549	ATCC VR-12
HAdV-B14	GZ01	A549	[22]
HAdV-B35	Holden	A549	ATCC VR-718
HAdV-B55	Shanxi-Y16	A549	[23]
HAdV-C1	Clinical isolate	A549	Unpublished
HAdV-C2	Clinical isolate	A549	Unpublished
HAdV-C5	Clinical isolate	A549	Unpublished
HAdV-E4	GZ01	A549	GenBank: KF006344.1
HAdV-F40	Dugan	HCT-8	ATCC VR-931
HAdV-F41	Tak	HCT-8	ATCC VR-930
H1N1	A/PR/8/34	MDCK	ATCC VR-95
H3N2	A/Aichi/2/68	MDCK	ATCC VR-1680
Influenza B virus (Inf B)	Victoria	MDCK	Unpublished
Respiratory syncytial virus A (RSV A)	Long	BHK-21	ATCC VR-26
Respiratory syncytial virus B (RSV B)	Clinical isolate	BHK-21	Unpublished
Enterovirus 71 (EV71)	GZ08	Vero	GenBank: FJ360545.1
Coxsackievirus A16 (CVA16)	GZ08	Vero	GenBank: FJ198212.1

### Whole blood sample

One adult human whole-blood sample was obtained from Guangzhou Blood Centre (Guangzhou, China) with serum indicating a high titer of neutralizing antibodies against HAdV-B3 and B7 that was validated using the microneutralization assay. This sample was used to create the Fab phage display libraries as described below.

### Construction of Fab phage display libraries

Fab phage display libraries were constructed using the pComb3X vector expression system based on a well-established protocol (Protocol 9.1: Human Fab Libraries)[24]. Peripheral blood mononuclear cells (PBMC) were isolated from fresh whole blood within 2 h after collection using the Lymphocyte Separation Medium (TBDsciences, Tianjin, China). Total RNA was extracted from PBMC using the *EasyPure* RNA Kit (Transgen, Beijing, China), and cDNA was synthesized from the total RNA sample using the *TransScript* II All-in-One First-Strand cDNA Synthesis SuperMix for PCR (Transgen, Beijing, China). To generate Fab gene segments, a three-step PCR method was performed using PrimeSTAR HS DNA Polymerase with GC Buffer (TaKaRa, Tokyo, Japan). The resultant Fab gene segments were digested with restriction enzyme *Sfi* I (New England Biolabs, Ipswich, MA, USA), and ligated into the phagemid pComb3XSS that had been cut with the same restriction enzyme using T4 DNA Ligase (New England Biolabs, Ipswich, MA, USA). Recombinant plasmids were transformed into competent *Escherichia coli* (*E*. *coli*) TG1 cells by electroporation (Bio-Rad, Hercules, CA, USA).

### Selection of HAdV-B3-binding phage through panning

Library biopanning procedures were carried out as described previously[25]. For this procedure, *E*. *coli* TG1 cells harboring the phagemid libraries were cultured to produce phage particles, which were rescued with helper phage M13KO7 (New England Biolabs, Ipswich, MA, USA). Amplified phage particles were subjected to three rounds of panning using immunotubes (Cat. no. 444202, Thermo Fisher Scientific, Waltham, MA, USA), which were prepared by coating with purified HAdV-B3 in 1× enzyme linked immunosorbent assay (ELISA) coating buffer (Solarbio, Beijing, China) at 4 °C overnight. Eluted phage particles from the final round of panning were used to re-infect *E*. *coli* HB2151 cells, and the cells were then plated on agar plates and incubated at 37 °C overnight. Single bacterial colonies were picked randomly and placed in 100 μL 2× TY medium supplemented with 1% glucose and 100 μg/mL ampicillin in U-bottom 96-well plates, which were incubated with shaking (250 rpm) at 37 °C overnight. A small inocula (about 2 μL) from the above plates were separately transferred to a second set of U-bottom 96-well plates containing 200 μL 2× TY supplemented with 0.1% glucose and 100 μg/mL ampicillin per well. These were grown with shaking (250 rpm) at 37 °C until the optical density of the culture at 600 nm (OD_600_) was approximately 0.9 (about 3 h). Then, 25 μL 2× TY supplemented with 9 mM isopropyl-β-d-thiogalactoside (IPTG) and 100 μg/mL ampicillin were added separately, and the cultures were further incubated with shaking (250 rpm) at 30 °C overnight. Supernatants (about 50 μL) used for screening were harvested separately by centrifugation at 1,800 *g* at 4 °C for 10 min.

### Screening of supernatants

Supernatant screening was performed based on the protocol described previously, except chemiluminescence enzyme immunoassay (CLEIA) was used in place of ELISA[25]. For supernatant CLEIA, immunoassay microplates (Cat. no. 463201, Thermo Fisher Scientific, Waltham, MA, USA) were prepared by coating with purified HAdV-B3 in 1× ELISA coating buffer at 4 °C overnight. Coated plates were then washed thrice with 0.05% Tween-20 in phosphate buffered solution (PBST) (Solarbio, Beijing, China). After blocking with 2% nonfat milk in PBS (MPBS) at 25 °C for 2 h, supernatants diluted in 2% MPBS (1:2 dilution) were added and incubated at room temperature for 1 h, followed by 6 washes with 0.05% PBST. Bound Fabs were detected by incubation with a 1:5000 dilution of horseradish peroxidase (HRP)-conjugated Protein L (GenScript, Piscataway, NJ, USA) in 2% MPBS at room temperature for 1 h, followed by 6 washes with 0.05% PBST. A solution of enhanced luminol-based chemiluminescent substrate (Transgen, Beijing, China) was added, and samples were assessed by a microplate luminometer (BioTek, Winooski, VT, USA). The photons of light emitted were measured as relative light units (RLU). The phagemid construct was isolated from the clone showing maximum binding, and the nucleotide sequences of the variable regions were determined by sequencing.

### Expression and purification of Fabs

Fab-secreting *E*. *coli* HB2151 cells selected by supernatant CLEIA were separated and further processed for Fab expression. Bacteria were inoculated in 1 L 2× TY medium supplemented with 1% glucose and 100 μg/mL ampicillin with shaking (250 rpm) at 37 °C until the culture reached an OD_600_ of approximately 1.0–1.2. Then, bacteria were harvested by centrifugation at 10,000 *g* at 25 °C for 20 min, and the pellet was re-suspended in 1 L super broth medium containing 0.1 mM IPTG and 100 μg/mL ampicillin with vigorous shaking at 250 rpm. After expression was allowed overnight at 25 °C, bacteria were harvested by centrifugation at 10,000 *g* at 4 °C for 30 min, and the supernatant was filtered through a membrane filter with a 0.22 μm pore size before being purified. The Fab in the filtered supernatant was purified using Protein L Resin (GenScript, Piscataway, NJ, USA) as per the manufacturer’s protocol. In brief, a column containing 2 mL Protein L Resin was equilibrated with 20 mL binding buffer (0.15 M NaCl, 20 mM Na_2_HPO_4_, pH 8.0). The filtered supernatant was allowed to pass through the column at a flow speed of 1 mL/min. The column was washed thoroughly with 120 mL of wash buffer (0.15 M NaCl, 20 mM Na_2_HPO_4_, pH 8.0) with a flow speed of 2 mL/min, and the bound Fab was eluted by 40 mL elution buffer (0.1 M glycine, pH 2.5). The purified Fab solution was further concentrated, and buffer exchange was conducted with PBS by ultrafiltration through a 30 kDa cutoff Amicon Ultra-15 centrifugal filter device (Millipore, Bedford, MA, USA), The Fab concentration was measured spectrophotometrically (NanoDrop 2000, Thermo Fisher Scientific, Waltham, MA, USA).

### Expression and purification of knob proteins

The codon-optimized genes of the knob proteins of HAdV-B3, E4, and C5 and a mutant knob protein containing the amino acid fragment (residues 70–90) of the HAdV-C5 knob protein replaced by that of HAdV-E4 (residues 71–91) were individually synthesized and subcloned into the vector pQE-30. After confirmation by sequencing, the correct plasmid was transformed into *E*. *coli* M15 cells. Bacteria were inoculated into 1 L LB medium supplemented with 100 μg/mL ampicillin and 50 μg/mL kanamycin, and incubated with vigorous shaking at 250 rpm at 37 °C. When the OD_600_ reached around 0.6, expression was induced by the addition of IPTG to a final concentration of 1 mM, and the culture was further incubated with shaking (250 rpm) at 18 °C overnight. Bacteria were then harvested by centrifugation at 10,000 *g* at 4 °C for 20 min. The pellet was re-suspended in non-denaturing lysis buffer containing 1 mg/mL lysozyme. *ProteinSafe* Protease Inhibitor Cocktail, EDTA-free (Transgen, Beijing, China) was added to the suspension at 1:1000 dilution. After incubation on ice for 30 min, the suspension was sonicated on ice. The sonicated product was centrifuged at 10,000 *g* at 4 °C for 20 min to obtain the clear extract, which was filtered through a membrane filter with a 0.22 μm pore size before being purified. The filtered extract was further purified with the BeyoGold His-tag Purification Resin (Beyotime Biotechnology, Shanghai, China) according to the manufacturer’s protocol. The knob protein concentration was measured spectrophotometrically (NanoDrop 2000, Thermo Fisher Scientific, Waltham, MA, USA).

### Sodium dodecyl sulfate-polyacrylamide gel electrophoresis (SDS-PAGE)

Purified Fabs were preheated at 100 °C for 10 min and were separated and resolved by 10% SDS-PAGE under denaturing and reducing conditions. Purified knob proteins with and without preheating (100 °C for 10 min) were separated and analyzed by 10% SDS-PAGE under denaturing and reducing conditions. Staining was performed using BeyoBlue Coomassie Blue Super Fast Staining Solution (Beyotime Biotechnology, Shanghai, China) for 1 h, and the reaction mixture was destained in distilled water overnight. SDS-PAGE was used to analyze the purity of Fabs and knob proteins.

### CLEIA

The binding capacity of each Fab to viruses and knob proteins was determined by CLEIA. Immunoassay microplates (Cat. no. 463201, Thermo Fisher Scientific, Waltham, MA, USA) were prepared by coating with purified viruses (10 μg/mL) and native knob proteins (2 μg/mL) of HAdV-B3, E4, and C5 (negative control) separately in 1× ELISA coating buffer at 4 °C overnight. After washing thrice with 0.05% PBST, coated plates were blocked with blocking buffer (BSA) (Sangon, Shanghai, China) at 25 °C for 2 h. Fab in general antibody dilution buffer (Sangon, Shanghai, China) (0.01 mg/mL) was separately incubated in triplicate in wells coated with viruses and knob proteins at 37 °C for 1 h, followed by 6 washes with 0.05% PBST. Wells containing a blank control (PBS) were included. Binding Fabs were detected by incubation with a 1:5000 dilution of HRP-conjugated Protein L (GenScript, Piscataway, NJ, USA) at 37 °C for 1 h, followed by 6 washes with 0.05% PBST. A solution of enhanced luminol-based chemiluminescent substrate was added, and samples were assessed by microplate luminometer. The photons of light emitted were measured as RLU.

### IFA

Generated Fabs found to react with several types of HAdV following CLEIA were separately used in immunofluorescence studies with different types of HAdV-infected cells. Each type of HAdV stock containing 100 TCID_50_/100 μL was separately inoculated in triplicate onto 96-well plates containing a culture of cell monolayers at 80% confluence. Two hours after infection, the cells were washed thrice with DMEM/F-12, and then maintained in DMEM/F-12 at 37 °C with 5% CO_2_. After 2 days, when cytopathic effects (CPE) appeared, the cells were fixed in Immunol Staining Fix Solution (Beyotime Biotechnology, Shanghai, China) at 25 °C for 1 h. After washing in PBS, the cells were permeabilized and blocked in QuickBlock Blocking Buffer for Immunol Staining (Beyotime Biotechnology, Shanghai, China) at 25 °C for 1 h, and then incubated at 25 °C for 1 h with Fab diluted in QuickBlock Primary Antibody Dilution Buffer for Immunol Staining (Beyotime Biotechnology, Shanghai, China) (0.01 mg/mL). After washing in 0.05% PBST, the cells were incubated for an additional hour with a 1:100 dilution of FITC-labeled anti-6×His tag antibody (Cat. no. ab1206, Abcam, Cambridge, MA, USA) in QuickBlock Secondary Antibody Dilution Buffer for Immunofluorescence (Beyotime Biotechnology, Shanghai, China), and then washed again in 0.05% PBST. The cells were observed under a fluorescence microscope (Leica, Wetzlar, Germany). Uninfected cells were used as a blank control.

### Western blot

The binding capacity of each Fab to knob proteins was determined by western blot. Ten micrograms of purified knob proteins were preheated (100 °C for 10 min) and then separated and analyzed by electrophoresis on 10% SDS-PAGE under denaturing and reducing conditions, and transferred onto polyvinylidene fluoride membranes. The membranes were then blocked with QuickBlock Blocking Buffer for Western Blot (Beyotime Biotechnology, Shanghai, China) overnight. After washing again with PBS, Fab (5 μg/mL) in QuickBlock Primary Antibody Dilution Buffer for Western Blot (Beyotime Biotechnology, Shanghai, China) was added and incubated at 25 °C for 2 h. After washing thrice with 0.05% PBST, bound Fab was detected by incubation with a 1:5000 dilution of HRP-conjugated anti-HA tag mouse mAb (Abbkin, Redlands, CA, USA) in QuickBlock Secondary Antibody Dilution Buffer for Western Blot (Beyotime Biotechnology, Shanghai, China) at 25 °C for 1 h, and washed thrice in 0.05% PBST. The blot was developed by the ECL Detection System (UVP Chemstudio, Analytik Jena AG, Thuringia, Germany).

### Multiple alignment of amino acid sequences of knob proteins

In order to identify the amino acid sequence of the linear epitope that was shared between HAdV-B3 and HAdV-E4 but differed from HAdV-C5, multiple alignment of the amino acid sequences of knob proteins of the indicated types was performed using the ClustalX software (version 2.1)[26] with the following parameters: gap opening, 10; gap extension, 0.2; delay divergent sequences, 30%. The Gonnet series was used for the protein weight matrix.

### Molecular modeling

The SWISS-MODEL workspace was used to model the three-dimensional (3D) structure of the knob protein of HAdV-E4[27]. The amino acid sequence of the HAdV-E4 knob protein was submitted to build a 3D model using the automated mode. Template selection, alignment and model building are completely automated by the server. The epitope region that was predicted to be exposed on the surface of the knob protein was selected as the potential site recognized by Fab.

## Results

### Fab libraries construction and supernatants screening

Two phage display libraries, named Fab_Kappa_ and Fab_Lambda_, were constructed successfully with capacities of 1.4×10^7^ and 2.45×10^7^, respectively. After three rounds of panning of the combined phage display Fab library, 384 individual clones were picked randomly and used to produce Fab, which was tested for binding to purified HAdV-B3 using supernatant CLEIA. Among those selected, four representative clones, which had supernatants that individually displayed the highest RLU in each 96-well immunoassay microplate, were analyzed by sequencing and BLAST analysis. The BLAST results showed that all clones originated from the Fab_Kappa_ library, and three of the four clones were identical. Finally, two unique clones, named 2A6 and 4E2, were selected.

### Fab expression and purification

**Fig 1:**
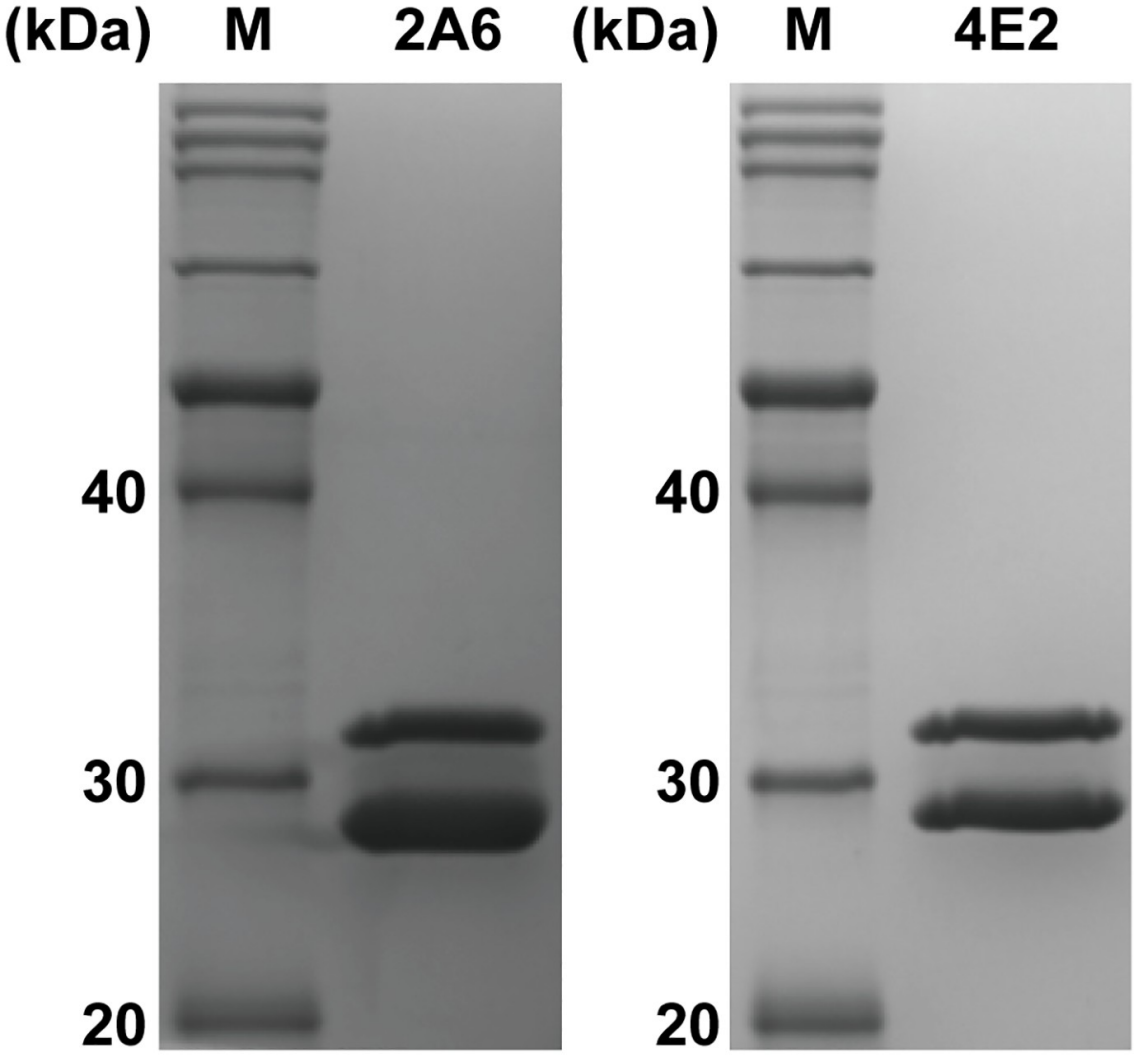
SDS-PAGE analysis of the purified Fabs. Purified Fabs 2A6 and 4E2 were preheated and then individually resolved by SDS-PAGE under denaturing and reducing conditions. The heterodimer was dissociated into the light chain (28 kDa) and the Fd fragment (32 kDa). Lane M represents the protein marker.

Fabs 2A6 and 4E2 were separately purified from the supernatants of bacterial cultures using protein L affinity purification. For both Fabs, 1 L of the bacterial culture typically yielded approximately 1 mg of the finial purified Fab product. The purified Fabs were individually verified by SDS-PAGE, which showed two bands corresponding to the predicted molecular weight of the light chain and the Fd fragment, respectively (Fig 1).

### Knob proteins expression and purification

**Fig 2:**
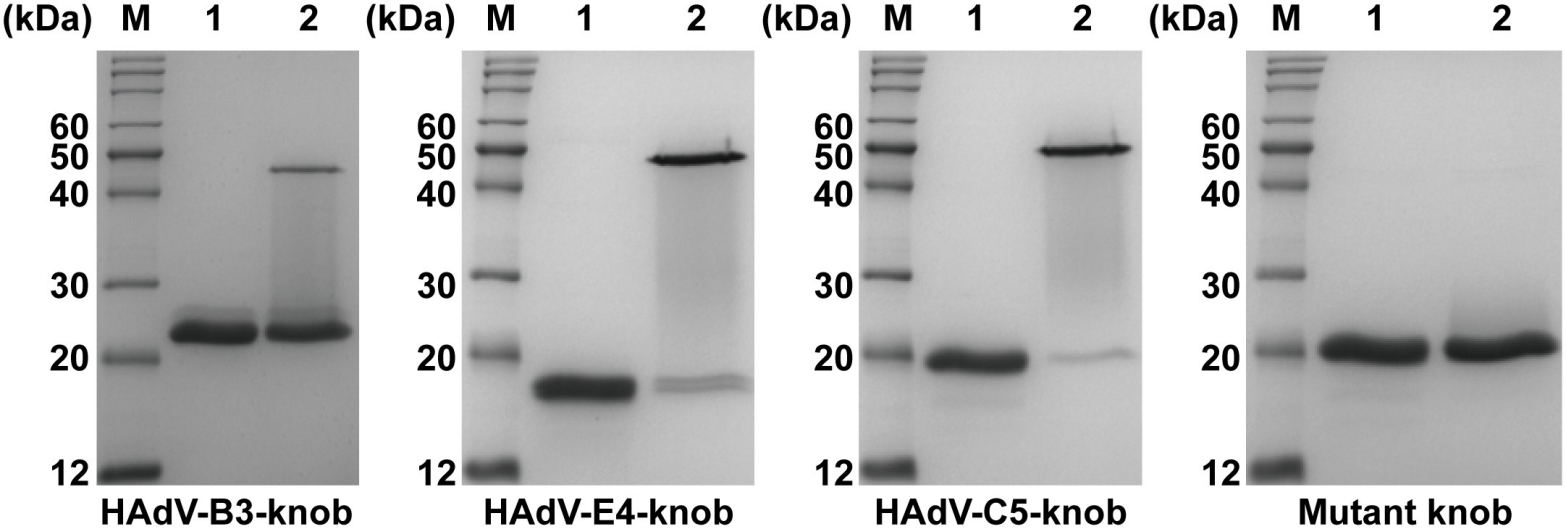
SDS-PAGE analysis of the purified knob proteins. The purified knob proteins of HAdV-B3, E4, and C5 and the mutant knob protein with and without preheating (lanes 1 and 2, respectively) were individually analyzed by SDS-PAGE under denaturing and reducing conditions. Coomassie Brilliant Blue staining of the purified knob proteins of HAdV-B3, E4 and C5 and the mutant knob protein with preheating revealed bands corresponding to the expected molecular weight of the monomeric proteins (23 kDa, 18 kDa, 20 kDa and 20 kDa, respectively). The purified knob proteins of HAdV-E4 and C5 without preheating were revealed in polymeric form, while the purified knob protein of HAdV-B3 and the mutant knob protein without preheating were revealed in monomeric form. Lane M represents the protein marker.

Purified knob proteins of HAdV-B3, E4, and C5 and the purified mutant knob protein were individually analyzed by 10% SDS-PAGE under denaturing and reducing conditions. Purified knob proteins that were preheated and separated by SDS-PAGE gel were stained with Coomassie Brilliant Blue, revealing bands corresponding to the expected molecular weight of the monomeric proteins (Fig 2). Under denaturing and reducing conditions, the knob proteins of HAdV-E4 and C5 that were purified without preheating were shown in polymeric form, but the purified HAdV-B3 knob protein and the mutant knob protein without preheating were in monomeric form (Fig 2). The result of SDS-PAGE showed that the purity of the knob proteins was over 95%.

### Virus-binding capacity

**Fig 3:**
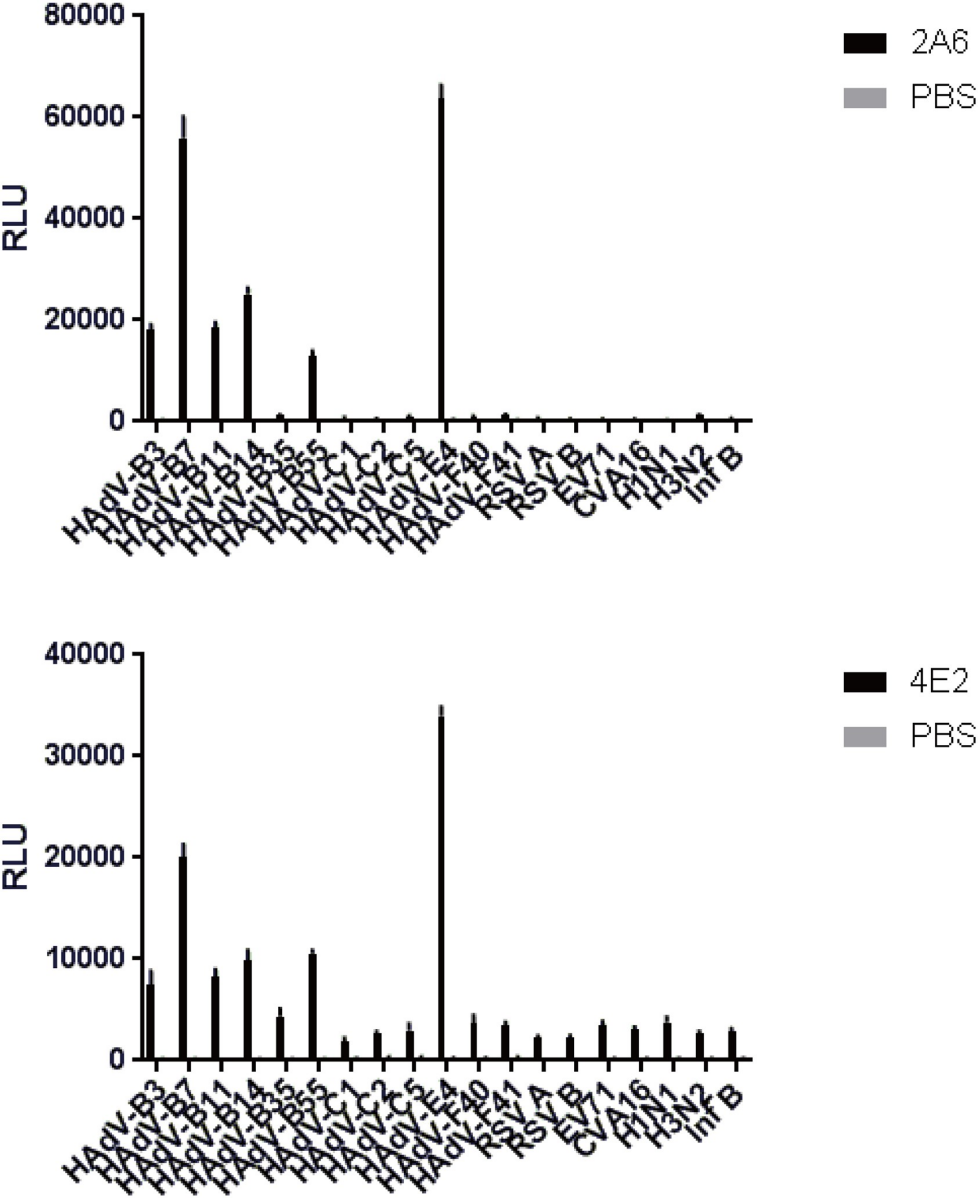
Measurement of virus-binding capacity of Fabs 2A6 and 4E2 using CLEIA. The virus-binding capacities of Fabs 2A6 and 4E2 were measured by separately binding to several types of HAdV, IFV, RSV, CVA16, and EV71. PBS was used as a blank control. The photons of light emitted were measured as RLU. Each virus was tested in duplicate, and the results represent the mean ± SD of three measurements. The average RLU of Fab 2A6 that reacted with HAdV-B35 was higher than those of the Fab that reacted with IFV, RSV, CVA16, and EV71. A similar result was found with Fab 4E2.

Compared with the other viruses, all RLU averages of Fab 2A6 that reacted with HAdVs were high (Fig 3). All RLU averages of Fab 2A6 that reacted with HAdVs in species B and E were higher than those of the Fab that reacted with those in species C and F, except HAdV-B35 (Fig 3). A higher average RLU of Fab 2A6 that reacted with HAdV-E4 was found (Fig 3). A similar result was found with Fab 4E2 (Fig 3). However, the results showed that Fab 2A6 displays a higher RLU than Fab 4E2 under the same experimental conditions (Fig 3).

### Immunofluorescence of HAdVs-infected cells

**Fig 4:**
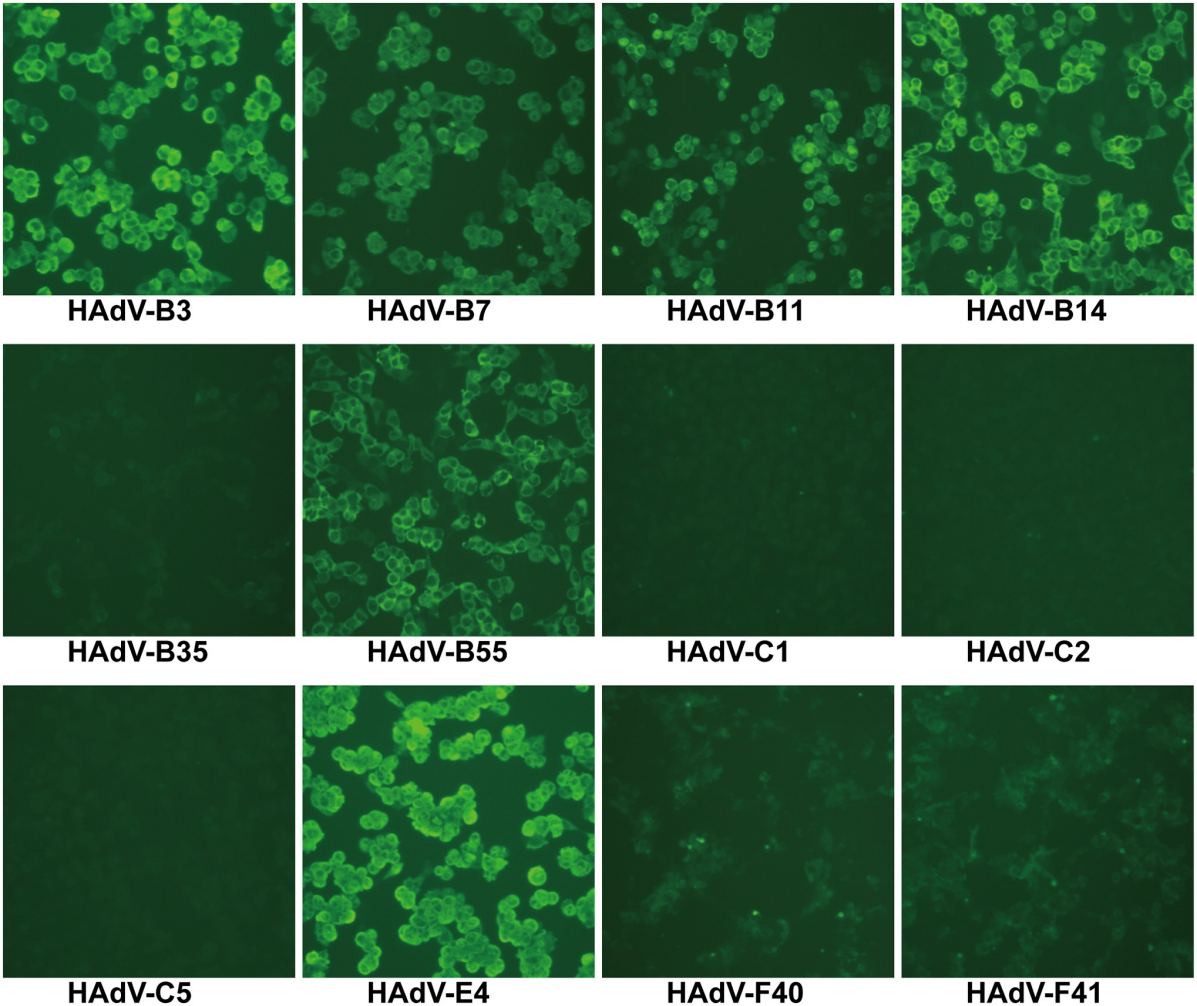
Immunofluorescence analysis of Fab 2A6 with different types of HAdV-infected cells. HAdV-B3, B7, B11, B14, B35, B55, C1, C2, C5, E4, F40, and F41 were individually used to examine Fab 2A6 with HAdV-infected cells using immunofluorescence. Cell cultures with obvious CPE were visualized. The cells were observed by a fluorescence microscope (100×).

**Fig 5:**
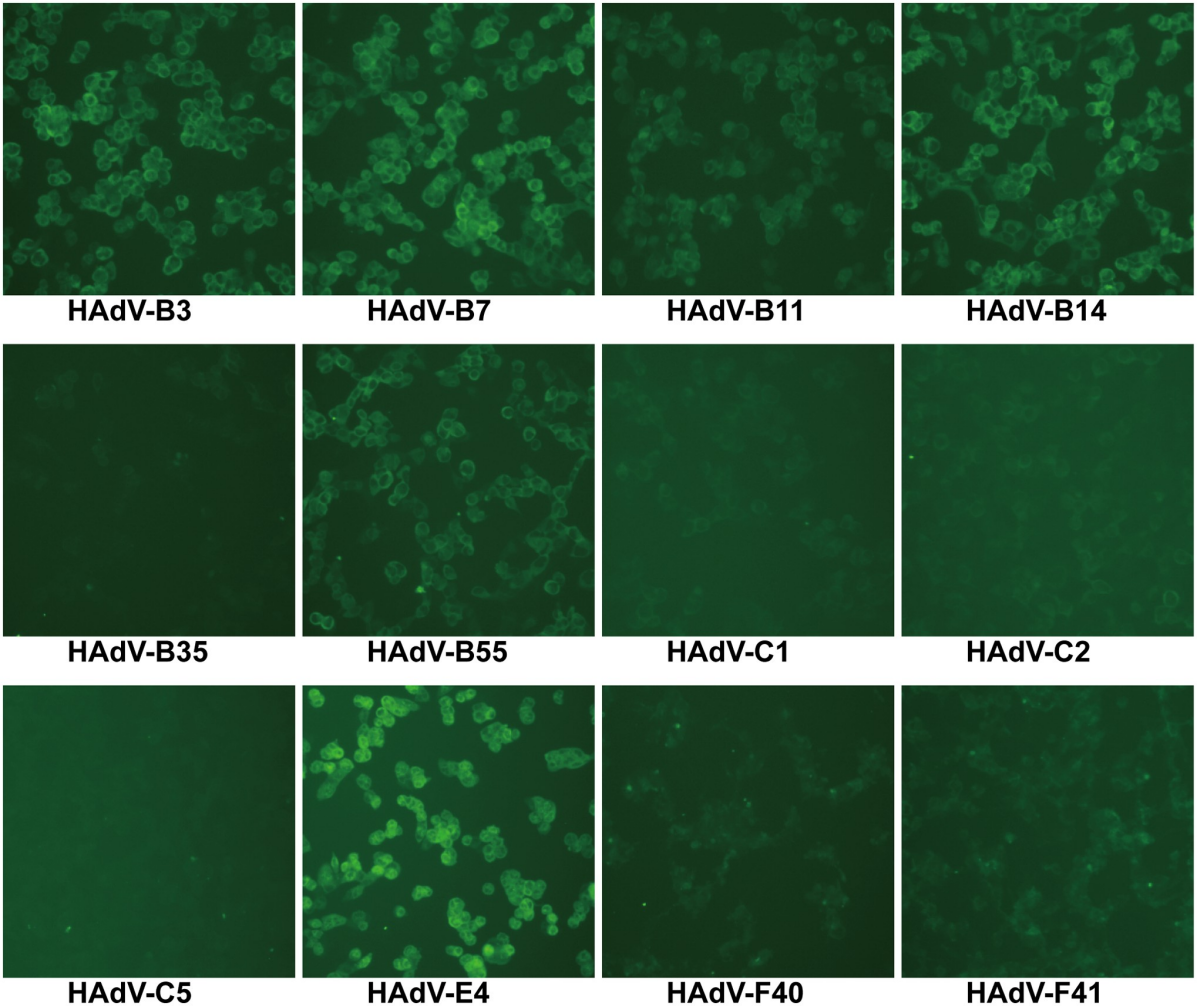
Immunofluorescence analysis of Fab 4E2 with different types of HAdV-infected cells. HAdV-B3, B7, B11, B14, B35, B55, C1, C2, C5, E4, F40, and F41 were individually used to examine Fab 4E2 with HAdV-infected cells using immunofluorescence. Cell cultures with obvious CPE were visualized. The cells were observed by a fluorescence microscope (100×).

When tested by IFA, Fabs 2A6 and 4E2 recognized cells infected with HAdV-B3, B7, B11, B14, B55, and E4 (Figs 4 and 5). No fluorescence was observed in cells infected with HAdV-B35, C1, C2, C5, F40, and F41 (Figs 4 and 5). As expected, both Fabs showed stronger fluorescence with A549 cells infected with HAdV-E4 than with other HAdVs (Figs 4 and 5). In immunofluorescence stained infected cells, both Fabs featured a clear cytoplasmic pattern against infected cells. The infected cells individually stained with Fabs showed that Fab 2A6 displays stronger fluorescence than Fab 4E2 under the same experimental conditions.

### Knob protein-binding capacity

**Fig 6:**
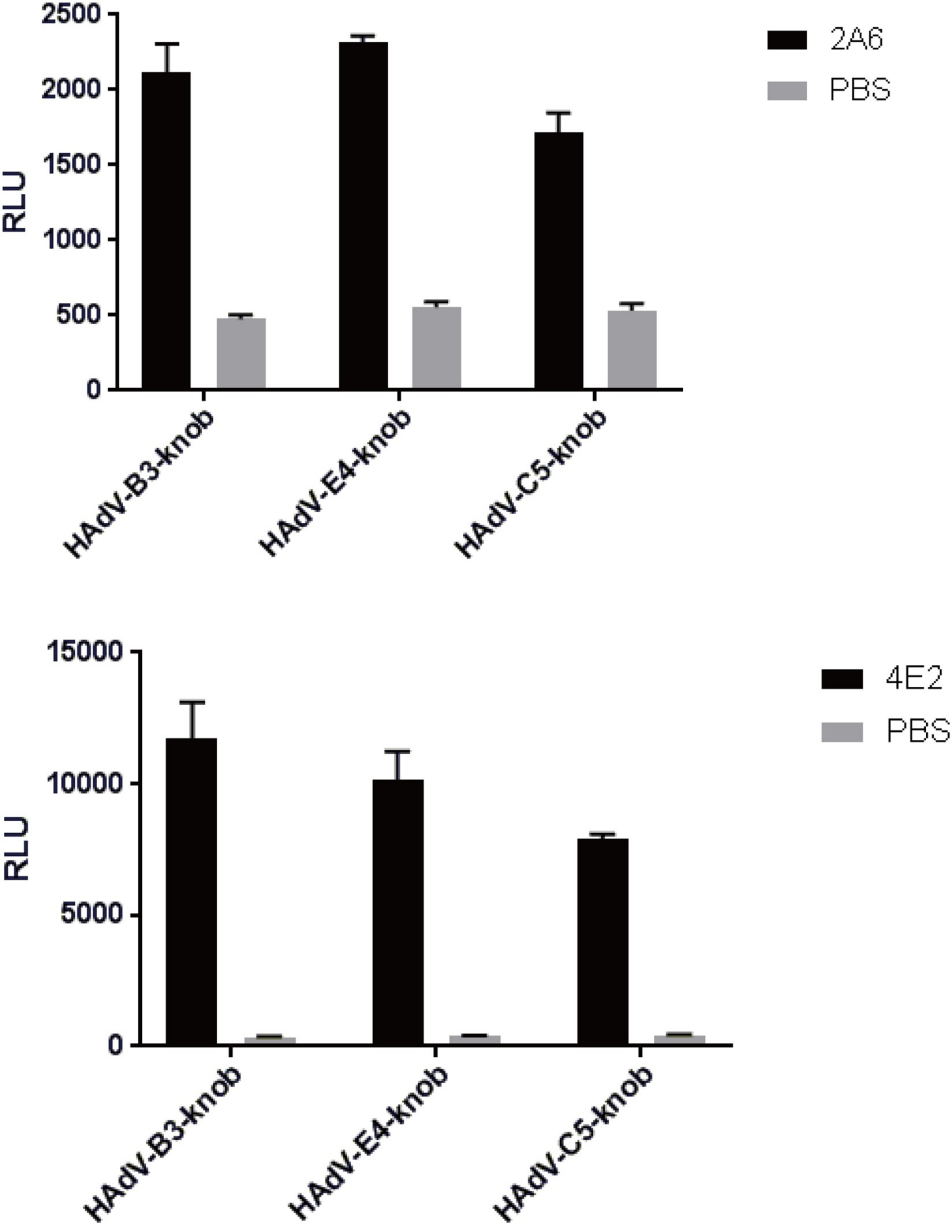
Measurement of knob protein-binding capacity of Fabs 2A6 and 4E2 using CLEIA. The knob protein-binding capacities of Fabs 2A6 and 4E2 were measured by binding to the knob proteins of HAdV-B3, E4, and C5 (negative control). PBS was used as a blank control. The photons of light emitted were measured as RLU. Each knob protein was tested in duplicate, and the result represents the mean ± SD of three measurements.

The average RLU of Fab 2A6 that reacted with the knob protein was higher in HAdV-B3 and E4 than in HAdV-C5 (negative control) (Fig 6). However, the average RLU of Fab 2A6 that reacted with the knob protein of HAdV-C5 was much higher than that of PBS (blank control) (Fig 6). A similar result was found with Fab 4E2. The average RLU of Fab 2A6 was higher than that of Fab 4E2 under the same experimental conditions (Fig 6).

### Epitope mapping

**Fig 7:**
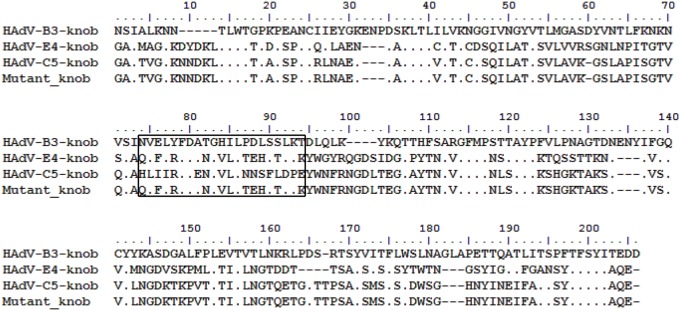
Multiple alignment of amino acid sequences of the knob proteins of HAdV-B3, E4, and C5 and mutant knob protein. The multiple alignment results showed that, compared with the sequences outside of the boxed region, the amino acid sequence (residues 71–91) of HAdV-E4 was found to be more similar to that of HAdV-B3 (residues 69–89) than to that of HAdV-C5 (residues 70–90) within the boxed region. The amino acid fragment of the HvdA-E4 knob protein within the boxed region was predicted to be the linear epitope. The mutant knob protein was designed by replacing the amino acid sequence of the HAdV-C5 knob protein within the boxed region with the amino acid sequence of the predicted linear epitope.

**Fig 8:**
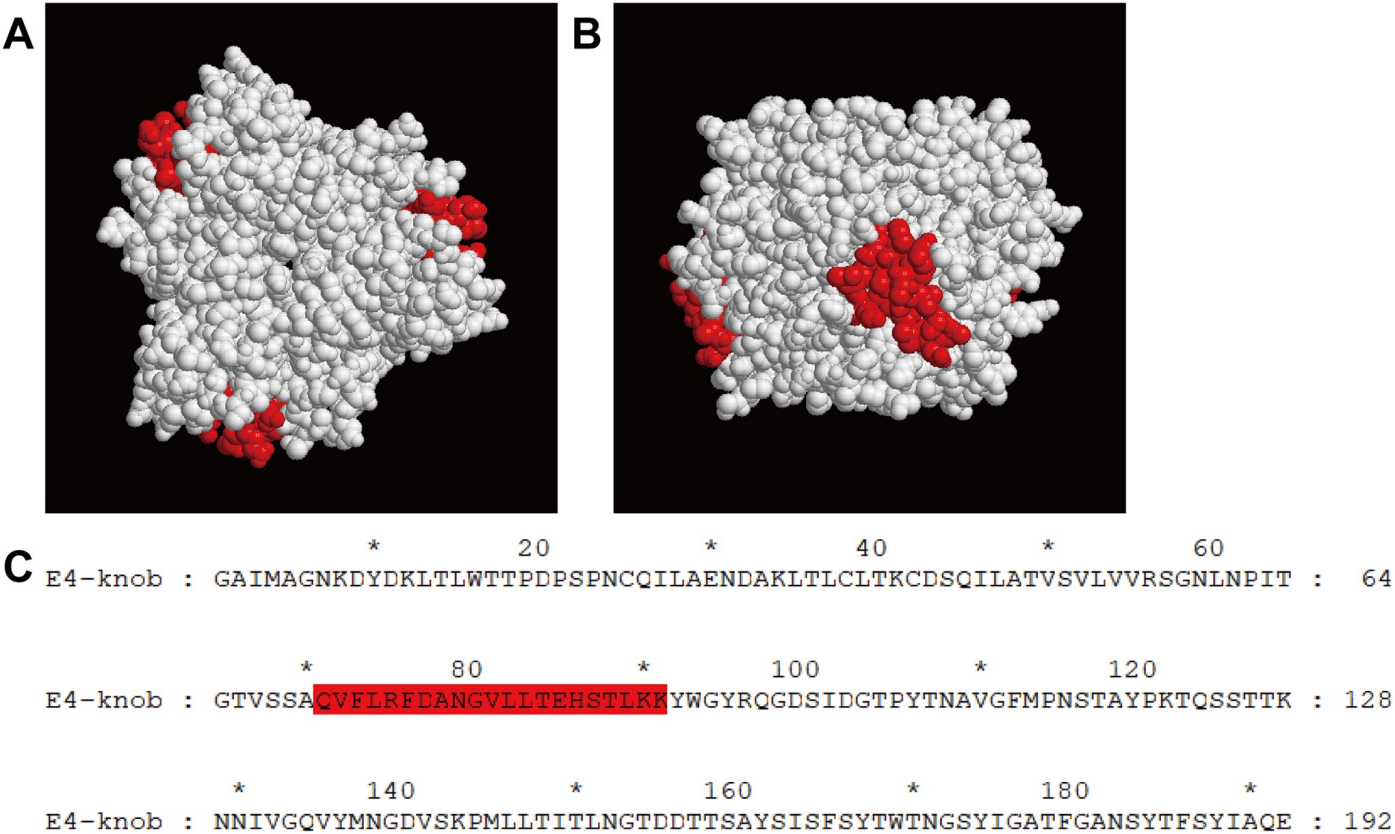
3D structure of the HAdV-E4 knob protein and the predicted linear epitope. The predicted linear epitope region on the HAdV-E4 knob protein is located in the neck region on the side of the 3D model. The predicted linear epitope region is highlighted in red on the model in both top (A) and side (B) views. The amino acid sequence of the predicted linear epitope is highlighted in red in the sequence of HAdV-E4 knob protein.

**Fig 9:**
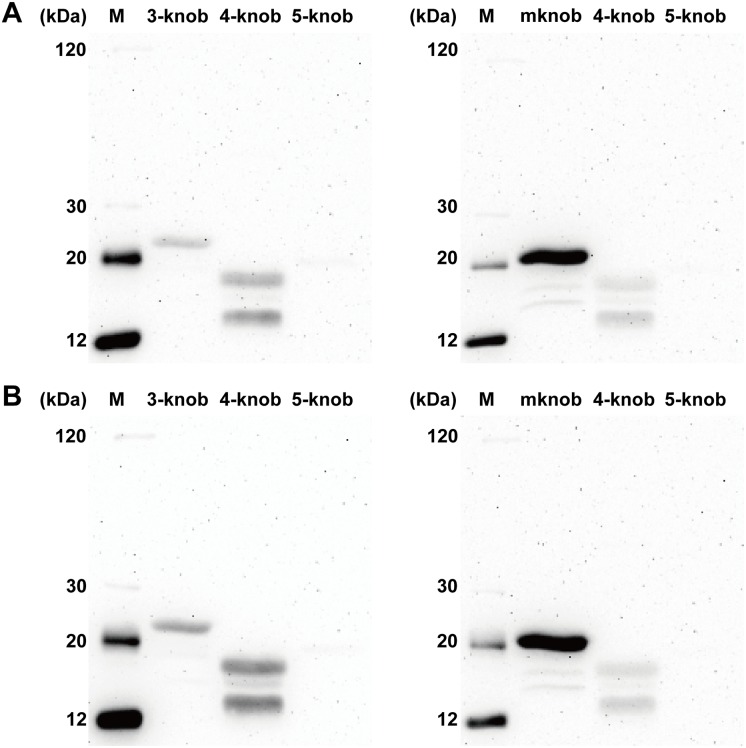
Western blot analysis of Fabs 2A6 and 4E2 binding to knob proteins under denaturing and reducing conditions. Note that Fabs 2A6 (A) and 4E2 (B) bind to the knob proteins of HAdV-B3 and E4 with molecular masses of about 23 kDa and 18 kDa, respectively, with preheating. However, both Fabs did not recognize the HAdV-C5 knob protein with preheating. In addition, a band around 20 kDa, which is the predicted molecular weight of the mutant knob (mknob) protein with preheating, was detected with both Fabs. Two non-specific bands appeared below the knob proteins of HAdV-B3 and E4, but these were not found with SDS-PAGE, suggesting that the concentrations were low. These non-specific proteins could be purified by Ni-NTA resin and recognized by Fab, suggesting that they contained the 6×His tag and the linear epitope. We inferred that these non-specific proteins were produced as partially expressed knob proteins that were degraded in *E*.*coli*.

After sequence alignment, the sequence of an amino acid fragment (residues 71–91) of HAdV-E4 was found to be more similar to the sequence in the same region (residues 69–89) of the HAdV-B3 knob protein with 47.62% homology than to that (residues 70–90) of HAdV-C5 with 38.1% homology (Fig 7). A model of the HAdV-E4 knob protein was successfully built based on the best template of the HAdV-C2 knob protein (PDB: 1qhv.1.A) with 65.26% homology (Fig 8). In further analyses, because this amino acid fragment (residues 71–91) was located outside of the 3D model, it was selected as the predicted linear epitope that could be recognized by Fab. Both Fabs detected the knob proteins of HAdV-B3 and E4 with preheating under denaturing and reducing conditions in western blot analysis, but neither Fab reacted with the knob protein of HAdV-C5 under the same conditions (Fig 9). In further analyses, both Fabs detected the mutant knob protein under these conditions (Fig 9).

## Discussion

Since there are many types of HAdV, mAbs employed in diagnostic reagents for HAdV detection should ideally target highly conserved epitope types in order to minimize the risk of failure in detecting new emergent types, as recombination is an accepted feature of HAdV evolution. In this study, Fabs 2A6 and 4E2 with specific reactivity to HAdV were developed using phage antibody library technology and evaluated for their applicability in developing IFA for HAdV detection. Both Fabs were expressed in *E*. *coli* at a relatively high level, and purified Fabs could be obtained in only 3 days. Compared with other expression systems, the *E*. *coli* expression system is more economical and straightforward for protein production. Based on the design of the vector pComb3XSS, the fusion tags 6×His and HA allow for purification and detection of Fab. In addition to these tags, both Fabs also can be purified and detected by Protein L. We investigated the cross-reactivity of both Fabs to different types of HAdV using CLEIA and IFA, and the results demonstrated that both Fabs strongly reacted with HAdV-B3, B7, B11, B14, B55, and E4. The results of CLEIA and IFA differed between both Fabs, indicating that the HAdV-binding capacity of Fab 2A6 was stronger than that of Fab 4E2. The specificity was examined by CLEIA using other respiratory viruses, such as influenza virus (IFV) and RSV. We found no cross-reactivity between either Fab and IFV or RSV. The specificity and cross-reactivity of both Fabs to HAdVs indicated that the generated Fabs could be applied in the development of diagnostic tests for HAdV. When the generated Fabs are used to detect antigens in nasopharyngeal swab samples, they are specific to HAdV and will not recognize other viruses, ensuring that the results are reliable. Some mAbs with specificity to HAdV have been reported, but they recognize only one or several types within a single species[16, 28, 29]. In this study, the generated Fabs recognized several types within species B and HAdV-E4. So far, no mAbs have been reported that recognize all known types of HAdV. The number of HAdV types that are recognized by a diagnostic reagent can be increased using a pool of two or more noncompeting antibodies. The number of mAbs used in the development of a diagnostic reagent for HAdV detection can be decreased using the generated Fabs, as both Fabs recognized at least six types of HAdV.

Fabs 2A6 and 4E2 were able to bind to several types of HAdV in species B and E, as assessed by CLEIA and IFA. We had recently reported that three broadly neutralizing mAbs were identified with specific reactivity to a conformational neutralization epitope in the knob proteins of several types of HAdV in species B[29]. Therefore, the knob proteins of HAdV-B3, E4, and C5 (negative control) were expressed to examine whether both Fabs also bind to the knob protein. Unfortunately, CLEIA determined that the binding of both Fabs to knob proteins was less specific. This may result from the structure of the native knob protein expressed by *E*. *coli* differing from that of the natural knob protein of HAdV. However, it seems that both Fabs bind to the purified knob protein of HAdV-B3, which was revealed by SDS-PAGE in monomeric form under denaturing and reducing conditions. Therefore, the binding ability of both Fabs to the knob proteins of HAdV-B3, E4, and C5 (negative control) in monomeric form was further examined. In western blot, both Fabs detected the knob proteins of HAdV-B3 and E4 with preheating, but they did not react with the knob protein of HAdV-C5 with preheating under denaturing and reducing conditions. This suggested that the Fabs recognized a conserved linear epitope that was similar between HAdV-B3 and HAdV-E4, but differed from that of HAdV-C5. An epitope region meeting the above condition and predicted to be exposed on the surface of the HAdV-E4 knob protein was selected as the potential site based on the results of sequence alignment and molecular modeling. To test this prediction, a mutant knob protein was constructed by replacing the amino acid fragment of the HAdV-C5 knob protein (residues 70–90) with that of HAdV-E4 (residues 71–91). In further analyses, both Fabs reacted with the mutant knob protein with preheating under denaturing and reducing conditions in western blot analysis, demonstrating that the binding epitope was mapped to the amino acid fragment of the HAdV-E4 knob protein (residues 71–91). Based on the sequence alignment results, the amino acid sequence of the epitope of HAdV-E4 is relatively conserved with that of HAdV-B3 in the same region, and 10 of 21 amino acids are identical with 47.62% homology.

Two different Fabs recognized the same epitope, suggesting that the epitope caused the body to produce at least two kinds of antibodies. Our findings are very important for the development of IFA to detect HAdV. Both Fabs exhibited no neutralization activity against the HAdVs listed in Table 1 (S1 Fig). These results were unexpected, demonstrating that both Fabs bind to the epitope that plays no role in the neutralization of HAdV.

## Supporting information

S1 Fig Measurement of the neutralization of Fabs 2A6 and 4E2 by microneutralization assay. To measure neutralization, 165 μL purified Fabs (0.2 mg/mL) were individually mixed with an equal volume of HAdV-E4 stock containing 200 TCID_50_/100 μL, and then the virus-serum mixtures were incubated for 1 h at 37 °C in 5% CO_2_. Subsequently, the virus-Fab mixtures were separately inoculated in triplicate onto 96-well plates containing a culture of A549 cell monolayers at 80% confluence. Two hours later, cells were washed thrice with DMEM/F-12 and then maintained in DMEM/F-12 at 37 °C in 5% CO_2_. Control wells containing only uninfected cells or virus were included. After 3 days, the cells were observed to evaluate the appearance of CPE. The cells were observed by a microscope (100×). Cell cultures with obvious CPE were visualized in wells with either Fab.
(TIF)

S1 File Gene sequences of Fabs 2A6 and 4E2. Complete sequences between the restriction sites of *Sfi* I.
(DOCX)
