# Supplementary figures and images for: Development of two antigen-binding fragments to a conserved linear epitope of human adenovirus and their application in immunofluorescence
Source: PLoS One. 2019 Jun 26;14(6):e0219091. doi: 10.1371/journal.pone.0219091 (PMC6594634; doi:10.1371/journal.pone.0219091)

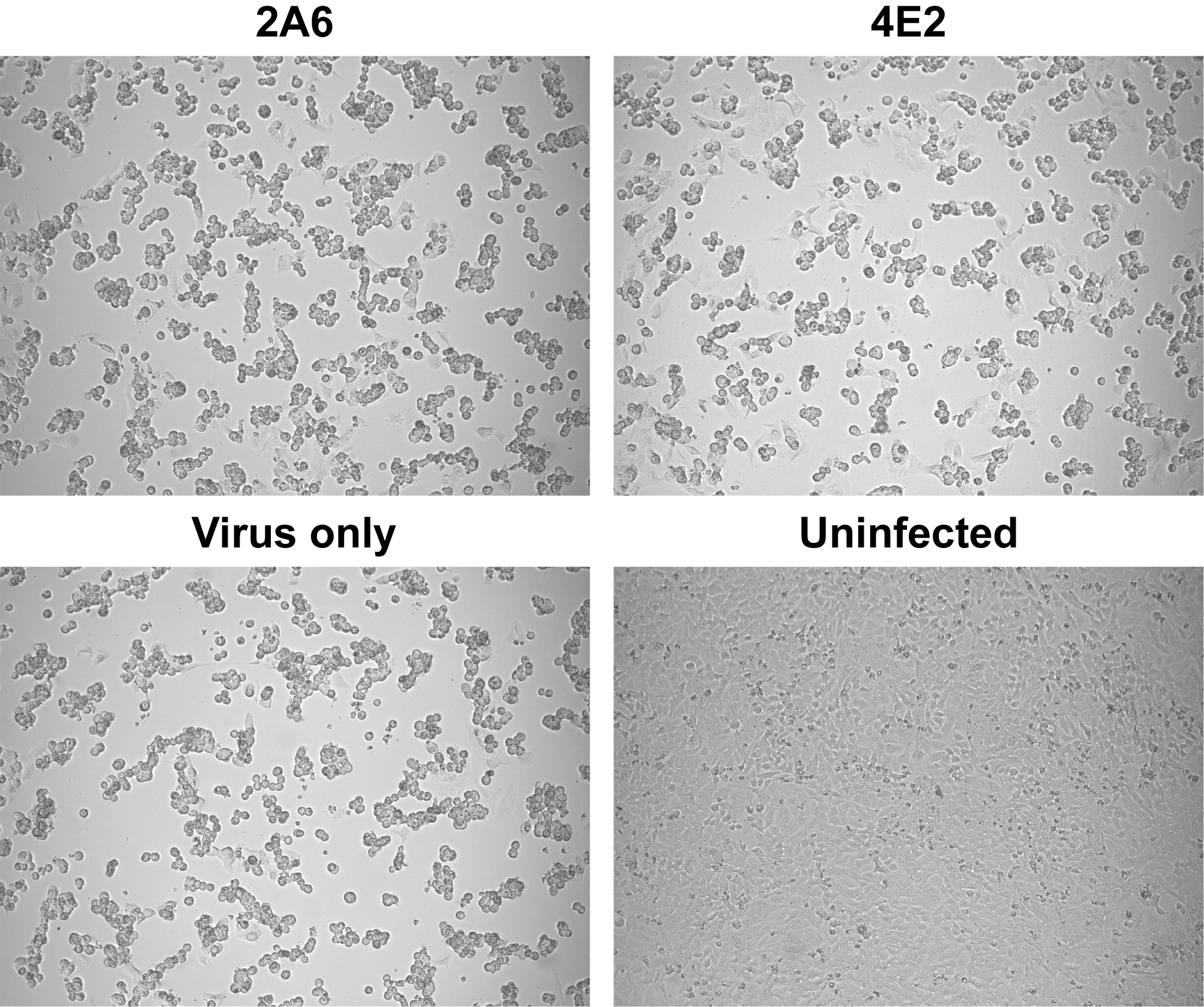

Supplement: S1 Fig — To measure neutralization, 165 μL purified Fabs (0.2 mg/mL) were individually mixed with an equal volume of HAdV-E4 stock containing 200 TCID50/100 μL, and then the virus-serum mixtures were incubated for 1 h at 37 °C in 5% CO2. Subsequently, the virus-Fab mixtures were separately inoculated in triplicate onto 96-well plates containing a culture of A549 cell monolayers at 80% confluence. Two hours later, cells were washed thrice with DMEM/F-12 and then maintained in DMEM/F-12 at 37 °C in 5% CO2. Control wells containing only uninfected cells or virus were included. After 3 days, the cells were observed to evaluate the appearance of CPE. The cells were observed by a microscope (100×). Cell cultures with obvious CPE were visualized in wells with either Fab. (TIF) [file pone.0219091.s001.tif]
