# Supplementary material for: Development of two antigen-binding fragments to a conserved linear epitope of human adenovirus and their application in immunofluorescence
Source: PLoS One. 2019 Jun 26;14(6):e0219091. doi: 10.1371/journal.pone.0219091 (PMC6594634; doi:10.1371/journal.pone.0219091)
Supplement: S1 File — Complete sequences between the restriction sites of Sfi I. (DOCX) [file pone.0219091.s002.docx]

>2H3

GAGCTCGTGATGACCCAGTCTCCATCCTCCCTGTCTGCATCTGTGGGGGACAGAGTCACCATCACTTGCCGGGCAAGTGAGAGCATAAGGGACCACTTAAATTGGTATCAAGTGACACCAGGGAAAGCCCCTAAGCTCCTGATCTCTGCTGCATCCAATTTGCAAAGTGGAGTCCCTCCGAGGTTCAGTGGCAGTGGATCTGGGACACAGTTCACTCTCACCATCAGCAGTCTGCAACCTGAAGATGTTGCGGATTACTTCTGTCAACAGAGTTACAGTACCCCAACTTTTGGCCAGGGGACCAACTTGGAGATCAAACGAACTGTGGCTGCACCATCTGTCTTCATCTTCCCGCCATCTGATGAGCAGTTGAAATCTGGAACTGCCTCTGTTGTGTGCCTGCTGAATAACTTCTATCCCAGAGAGGCCAAAGTACAGTGGAAGGTGGATAACGCCCTCCAATCGGGTAACTCCCAGGAGAGTGTCACAGAGCAGGACAGCAAGGACAGCACCTACAGCCTCAGCAGCACCCTGACGCTGAGCAAAGCAGACTACGAGAAACACAAAGTCTACGCCTGCGAAGTCACCCATCAGGGCCTGAGCTTGCCCGTCACAAAGAGCTTCAACAGGGGAGAGTGTTAGTTCTAGATAATTAATTAGGAGGAATTTAAAATGAAATACCTATTGCCTACGGCAGCCGCTGGATTGTTATTACTCGCTGCCCAACCAGCCATGGCCCAGGTGCAGCTGCAGGAGTCGGGCTCAGGACTGGTGAAGCCTTCACAGACCCTGTCCCTCAGCTGCTCTGTCTCTGGCGACTACATCACCAGTGGTGGTTACTACTGGAGCTGGATCCGGCAGCCAGTAGGGAAGGGCCTGGAGTGGATGGGGCACATCTTACATAGTGGGAGGACCTACTACAACCCGTCCCTCAAGAGTCGAGTCACCATGTCACTAGACAGGTCCAAGAACCAGGTCTCGCTGAAGCTGACCTCTGTGACCGCCGCGGACACGGCCGTGTATTTCTGTGCCAGAGCCCGACCCGACTCTGATTACTATGATCGTAGTGGTTCTTATCTTTACTACAATGACTACTGGGGCCAGGGAACCCTGGTCACCGTCTCCCCTGCCTCCACCAAGGGCCCATCGGTCTTCCCCCTGGCACCCTCCTCCAAGAGCACCTCTGGGGGCACAGCGGCCCTGGGCTGCCTGGTCAAGGACTACTTCCCCGAACCGGTGACGGTGTCGTGGAACTCAGGCGCCCTGACCAGCGGCGTGCACACCTTCCCGGCTGTCCTACAGTCCTCAGGACTCTACTCCCTCAGCAGCGTGGTGACCGTGCCCTCCAGCAGCTTGGGCACCCAGACCTACATCTGCAACGTGAATCACAAGCCCAGCAACACCAACGTGGACAAGAAAGTTGAGCCCAAATCTTGTGACAAAACTAGT

>4E2

GAGCTCGTGTTGACGCAGTCTCCAGCCACCCTGTCTTTGTCTCCAGGGGAAAGAGCCACCCTCTCCTGTAGGGCCAGTCAGAGTGTCGACAGCTACCTAGCCTGGTATCAACAAAAACCTGGCCAGGCTCCCAGGCTCCTCATCTATGATGCGTCCACCACGGCCCCTGGCATCCCAGCCAGGTTCACTGGCAGTGGGTCTGGGACAGACTTCACTCTCACCATCAGCGGCCTAGAGCCTGAAGATTTTGCAGTTTATTACTGTCAGCAGCGTTCCGGCTGGCCTCCTGTGCTCTCTTTCGGCGGAGGGACCAGGCTGGAGATCAAACGAACTGTGGCTGCACCATCTGTCTTCATCTTCCCGCCATCTGATGAGCAGTTGAAATCTGGAACTGCCTCTGTTGTGTGCCTGCTGAATAACTTCTATCCCAGAGAGGCCAAAGTACAGTGGAAGGTGGATAACGCCCTCCAATCGGGTAACTCCCAGGAGAGTGTCACAGAGCAGGACAGCAAGGACAGCACCTACAGCCTCAGCAGCACCCTGACGCTGAGCAAAGCAGACTACGAGAAACACAAAGTCTACGCCTGCGAAGTCACCCATCAGGGCCTGAGCTTGCCCGTCACAAAGAGCTTCAACAGGGGAGAGTGTTAGTTCTAGATAATTAATTAGGAGGAATTTAAAATGAAATACCTATTGCCTACGGCAGCCGCTGGATTGTTATTACTCGCTGCCCAACCAGCCATGGCCCAGGTGCAGCTGCAGGAGTCGGGCCCAGGACGAGTGAAGCCTTCGGAGACCCTGTCCCTCACCTGCACTGTTTCAGGTGGCTCCGTCAGTGGAGACTACTGGAGCTGGATCCGGCAGCCACCAGGGAAGGGACTGGAGTGGTTAGGGAATGTCTATTACGGTGGGTACACCAACTACAACCCATCCCTCAAGAGTCGAGTTTCCATGTCTGTAGACGCGTCCAAGAGGCAGGTCTCCCTGAGGATGACCTCTTTGACCCCTGCGGACACGGCCGTGTATTTCTGTGCGAGAAAGCCAAGTTACTTCGGACAGTTCACTGATTGGTACTATGACCAGTGGGGCCAGGGCACCCTGGTCACCGTCTCCTCTGCCTCCACCAAGGGCCCATCGGTCTTCCCCCTGGCACCCTCCTCCAAGAGCACCTCTGGGGGCACAGCGGCCCTGGGCTGCCTGGTCAAGGACTACTTCCCCGAACCGGTGACGGTGTCGTGGAACTCAGGCGCCCTGACCAGCGGCGTGCACACCTTCCCGGCTGTCCTACAGTCCTCAGGACTCTACTCCCTCAGCAGCGTGGTGACCGTGCCCTCCAGCAGCTTGGGCACCCAGACCTACATCTGCAACGTGAATCACAAGCCCAGCAACACCAAGGTGGACAAGAAAGTTGAGCCCAAATCTTGTGACAAAACTAGT
